# Perspectives on the Lindman Hypothesis and Cellulose Interactions

**DOI:** 10.3390/molecules28104216

**Published:** 2023-05-21

**Authors:** Magnus Norgren, Carolina Costa, Luís Alves, Alireza Eivazi, Christina Dahlström, Ida Svanedal, Håkan Edlund, Bruno Medronho

**Affiliations:** 1 Surface and Colloid Engineering, FSCN Research Centre, Mid Sweden University, SE-851 70 Sundsvall, Sweden; carolina.costa@miun.se (C.C.); alireza.eivazi@miun.se (A.E.); christina.dahlstrom@miun.se (C.D.); ida.svanedal@miun.se (I.S.); hakan.edlund@miun.se (H.E.); 2 Department of Chemical Engineering, CIEPQPF—Chemical Processes and Forest Products Engineering Research Centre, University of Coimbra, Pólo II–R. Silvio Lima, 3030-790 Coimbra, Portugal; luisalves@ci.uc.pt; 3 MED—Mediterranean Institute for Agriculture, Environment and Development, CHANGE—Global Change and Sustainability Institute, Faculdade de Ciências e Tecnologia, Universidade do Algarve, Campus de Gambelas, Ed. 8, 8005-139 Faro, Portugal

**Keywords:** cellulose, amphiphilicity, intermolecular interactions, dissolution, regeneration, emulsification, composite materials

## Abstract

In the history of cellulose chemistry, hydrogen bonding has been the predominant explanation when discussing intermolecular interactions between cellulose polymers. This is the general consensus in scholarly textbooks and in many research articles, and it applies to several other biomacromolecules’ interactions as well. This rather unbalanced description of cellulose has likely impacted the development of materials based on the processing of cellulose—for example, via dissolution in various solvent systems and regeneration into solid materials, such as films and fibers, and even traditional wood fiber handling and papermaking. In this review, we take as a starting point the questioning of the general description of the nature of cellulose and cellulose interactions initiated by Professor Björn Lindman, based on generic physicochemical reasoning about surfactants and polymers. This dispute, which became known as “the Lindman hypothesis”, highlights the importance of hydrophobic interactions in cellulose systems and that cellulose is an amphiphilic polymer. This paper elaborates on Björn Lindman’s contribution to the subject, which has caused the scientific community to revisit cellulose and reconsider certain phenomena from other perspectives.

## 1. Introduction

In 2010, a paper by Lindman, Karlström and Stigsson discussed the mechanisms of cellulose dissolution, pointing out some discrepancies in the common literature, where hydrogen bonding was argued as the most crucial interaction to overcome. The paper (re)introduced hydrophobic interactions and cellulose amphiphilicity as essential to consider for the successful development of future cellulose solvents [1]. In a review by Medronho and Lindman et al., published two years later, cellulose solubility or insolubility in water was revisited more carefully [2]. By considering some fundamental polymer physicochemical principles and some widely recognized inconsistencies in cellulose’s behavior, the authors emphasized that the hydrophobic molecular interactions have been underestimated, relative to hydrogen bonding, and are significantly important for the understanding of cellulose. Cellulose amphiphilicity was highlighted again. This time, one of the journal editors brought the matter into the spotlight for serious scrutinization by the scientific community. In a follow-up response paper, published in the same journal issue, several well-reputed cellulose scientists with a wide range of experience and representing a variety of scientific disciplines were invited to debate what was coined as “the Lindman hypothesis” [3]. Since then, a myriad of studies have been published dealing with cellulose dissolution and regeneration, and the consequences of cellulose’s amphiphilicity. This review, fully dedicated to the subject, is intended to highlight some important developments in the area over the years, based on what has been triggered by Lindman’s dispute, and give an outlook of emerging cellulose applications.

## 2. The “History” of Cellulose in Science and Technology

### 2.1. Addressing Cellulose Intermolecular Interactions

#### 2.1.1. In Cellulose Biosynthesis

Via freeze-fracture techniques applied in electron microscopy studies, cellulose biosynthesis by plasma membrane-bound complexes has been visualized. The first observations of the synthesis machinery were made at the tip of elongating cellulose microfibrils in the green alga *Oocystis*, and, due to this, the synthesis machinery was designated as the terminal complex (TC) [4]. In most algae, the TCs are organized as linear arrays, but in higher plants, the cellulose-synthesizing machinery is designated as rosettes and occurs in the form of hexagonal structures with six-fold symmetry [5,6]. Cellulose biosynthesis is a complex, cell-directed event. The nature of the enzyme complexes controls the outcome, essentially via the number and positioning of the glycosyltransferases within the complexes [7]. This thereafter leads to cellulose secretion to the cell surface through complex secretion pore structures.

Polymerization and crystallization are two separate and sequential steps in native cellulose biosynthesis [8]. It is known that the crystallization step is the rate-limiting step, and when crystallization is prevented by the binding of fluorescent brighteners, the polymerization rate can significantly increase. In biosynthesis, cellulose crystallization has been described as a three-step process, starting with the formation of monomolecular glucan chain sheets due to van der Waals forces. Thereafter, the association of these sheets leads to mini-crystals (sub-elementary fibrils) stabilized by H-bonding, and finally the convergence of the mini-crystals into the native crystalline microfibril takes place [9,10,11].

#### 2.1.2. In Dissolution and Regeneration of Cellulose

Currently, two processes in the dissolution and regeneration of cellulose are of technical and commercial importance: the viscose process and the lyocell process. The manufacturing of viscose rayon from cellulose raw material is based on an invention by Cross, Bevan and Beadle in 1891. The treatment involves dissolving pulps (sulfite and prehydrolysis kraft pulps) or cotton with concentrated alkali (NaOH), followed by a reaction with carbon disulfide (xanthation), which makes the intermediate soluble in NaOH (aq) and possible to process by spinning, casting and regeneration into fibers (rayon) and films (cellophane). Another type of regenerated cellulose fiber is lyocell (U.S. brand name Tencel). In the lyocell process, the cellulose solution is produced from dissolving pulp using the *N*-methylmorpholine-*N*-oxide (NMMO) solvent. The lyocell fiber is precipitated from NMMO, in which no substitution of the hydroxyl groups occurs, and no chemical intermediates are formed. The invention appeared first in a patent in 1981 by McCorsley, describing the basic process of dissolving cellulose [12]. Nowadays, Lenzing is the world’s largest lyocell fiber manufacturer, capable of supplying ca. 130,000 metric tons of lyocell fiber for the global rayon market each year.

**Figure 1:**
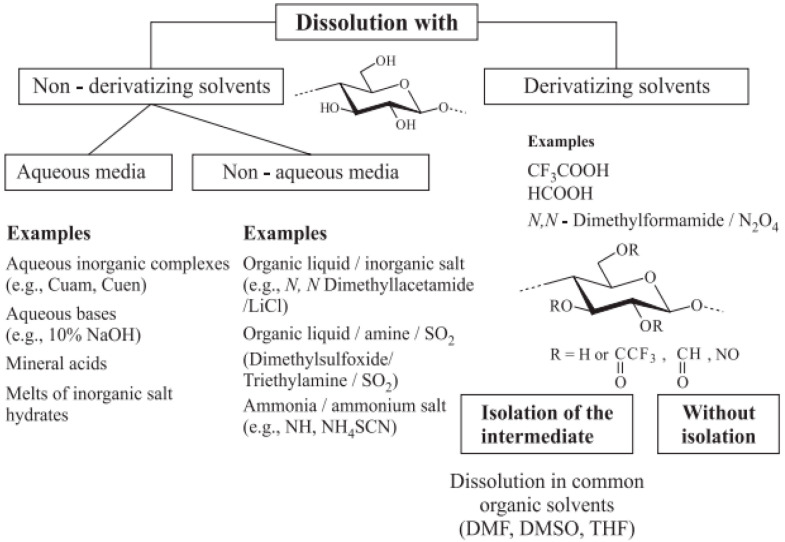
Classification of different cellulose solvents. Reprinted from [15].

Cellulose is insoluble in water and in hydrocarbons, but soluble in several simple and “exotic” solvents. Thus, non-derivatizing solvents for cellulose show enormous variation, including, e.g., strongly acidic and alkaline aqueous systems; aqueous NaOH and urea; transition metal complexes, such as copper salts mixed with concentrated ammonia (Cuoxam); aqueous copper–ethylenediamine complex solutions (CED); mixtures of dimethylsulfoxide with metal salts; *N*,*N*-dimethylacetamide (DMAc)/LiCl, NMMO; concentrated ZnCl_2_ aqueous solutions; and a range of different ionic liquids (IL) (Figure 1). In recent reviews, many additional examples are given and here also the significant diversity of solvents is underlined [13,14]. From a thermodynamic point of view, the formation of extended crystalline regions in cellulose implies the lowered solubility of these regions compared with the amorphous ones, since the crystalline state always has the lowest free energy.

#### 2.1.3. In Cellulose Swelling

**Figure 2:**
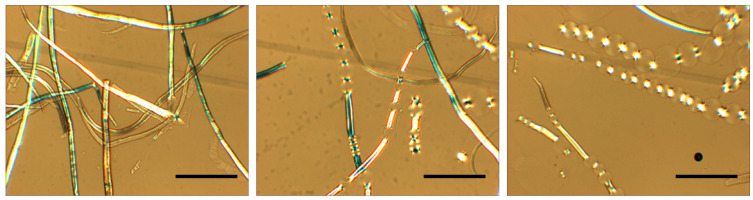
Nonhomogeneous swelling (ballooning phenomenon) in cellulose fibers when dispersed in cold aqueous-based alkali. The scale bar represents 100 μm.

Due to their complex hierarchical structures and organization, cellulose fibers show a different picture, characterized by heterogeneous swelling and dissolution [16]. The heterogeneous swelling can elicit unusual effects, such as the ballooning that occurs due to preferential swelling in specific areas along the fibers (Figure 2). In 1864, Nägeli described the ballooning phenomenon [17], and it was further reported in investigations by Pennetier [18], Flemming and Thaysen [19,20], Rollins and Tripp [21], Hock [22] and Warwicker et al. [23]. Later studies have shown that the swelling and dissolution mechanisms are strongly coupled with the solvent quality [24,25].

As described above, cellulose swelling in strongly alkaline solutions has been known for a long time, and the swelling is often accompanied, to some extent, by dissolution. In the groundbreaking work by Neale, this was attributed to the osmotic pressure of the counterions, as cellulose is deprotonated and charged at a high pH [26]. It was further discovered that, along with swelling at high hydroxide concentrations, there might be appreciable swelling also at a lower pH due to the occurrence of acidic groups, especially carboxylic acid groups, formed during wood pulp processing through hydrogen peroxide bleaching [27,28]. Moreover, sulfonic acid groups from the chemi-thermomechanical or sulfite pulping processes may also contribute to this. Furthermore, Lindström and Carlsson noted that the water retention values of holocellulose and unbleached sulfate pulps showed major increases as a function of pH, in the range in which carboxylic acid groups ionize [27]. Clearly, the osmotic swelling due to counterion entropy, notably in polymer gels, is due to the ionization of the polysaccharides and lignin, e.g., due to the dissociation of carboxylic acid groups in cellulose and hemicelluloses (and sulfonic acid and/or phenolic groups in lignin). As for polymer systems in general, swelling increases with the charge density and decreases with the electrolyte concentration and the valency of the counterions [29,30]. We will return to the pH effect in Section 3.2.

Regarding the combination of hydrophobic and electrostatic interactions in cellulose, the addition of thiourea produces enhanced cellulose swelling in NaOH solutions, as was reported by Zhang et al. [31]. Strong effects of electrolyte additions, where the difference in polarity between the two ions is significant, can also be expected to promote swelling—namely, combinations of high-charge-density cations, such as Ca^2+^ and Li^+^, with large, polarizable anions, such as I^−^ and SCN^−^. For example, LiSCN is very effective in enhancing swelling [32], which can be interpreted as the weak association of the anions with cellulose, whereas the cations are depleted. Furthermore, the significant swelling of cellulose fibers has been demonstrated using mixed solutions of NaSCN and urea [16,33].

#### 2.1.4. In Partial Dissolution and Plasticization of Cellulose

Plasticization can in some ways be regarded as extreme swelling, gelation or the partial dissolution of cellulose fibers, which drastically increases the cellulose chain’s mobility. Efficient plasticizing solvents should have similar properties to good dissolving agents. In this respect, the early findings of urea as a plasticizing agent were vital, showing that the weakening of the hydrophobic interactions between cellulose molecules has a key role [34]. Plasticized, or vulcanized, paper was already developed in the 1860s, when several layers of paper, impregnated by zinc chloride, capable of swelling the cellulose fibers and partially dissolving them, were pressed together and zinc chloride washed out in several steps [35]. Plasticization increases the density, mechanical strength and strain at the break of the paper, with an unchanged or slightly increased specific stiffness [36,37]. With improved mechanical properties, the range of uses for cellulose fiber products is expanded, capable of replacing plastics in many applications.

Plasticization partly changes the crystallinity of cellulose fibers from cellulose I to cellulose II and increases the content of amorphous cellulose, which thereby influences the properties of the fibers and paper [38]. Furthermore, plasticization increases the fiber-to-fiber bond strength. The surface layers of the cellulose fibers can also be affected, and higher strain at break, stiffness and tensile strength can be introduced [39,40,41].

#### 2.1.5. In Cellulose Pulp Fiber and Papermaking

Since ancient times, paper has been produced from fibers that were initially obtained via the processing of annual plants. Thanks to cellulose, correctly processed plants hold the fibrous structure needed to form a sufficiently strong network that can be dewatered, pressed and finally dried into sheets for further utilization. In the early days, the mechanical processing of plant raw material into lignocellulosic pulp, mainly for use as printing paper, was the only alternative. During industrialization in the 19th century, the mechanical processing of wood into fibers by “disassembling” logs with a stone grinder, often driven by waterpower, into groundwood pulp was introduced. Slightly later, chemical pulping was invented and evolved rapidly.

In its dried state, wood consists of polysaccharides and polyaromatic lignin, giving the wood a yellowish to brownish color. Somewhat dependent on the wood species, about 70% of the dry weight is constituted by the polysaccharides, and hereof cellulose is the main component [42]. In chemical pulping, most of the lignin is removed from the fibers and the superior brightness stability of the paper can be obtained after subsequent bleaching. The removal of lignin from the cellulose pulp fibers also makes them more flexible and adaptable when forming a web, and the produced paper material generally becomes much stronger. Thus, delignification brought new possibilities to utilize cellulose fibers in areas beyond printing paper, which were later exploited during the 20th century.

Due to the versatility of paper and its economic importance for many countries, a respectable amount of academic and industrial research has been dedicated to the topic over the years. A significant focus has been placed on developing and understanding the paper strength and the influence of fiber–water interactions [43]. The strength of the paper is obviously necessary for the final product, but, often, it is even more critical in paper production. The production rate in a paper machine can be far above 1000 m/min, and the forces exhibited under production can easily be detrimental and result in paper breaks and lost production. Of course, the different unit operations in the paper production process must be optimized as well. However, the paper strength from the wet to the dry state is finally the limiting parameter.

There is no doubt that hydrogen bonding between the fibers in paper has some importance for the strength, especially when no chemical additives are added into the papermaking stock. However, the beneficial effects of hydrogen bonding in the fiber–fiber interactions and paper strength are strictly limited to dried paper. If water surrounds the fibers, other phenomena due to, e.g., morphological changes related to fibrillation, and the geometrical and mechanical properties of the fibers and fiber walls introduced by directed processing, are always much more relevant to discuss [43]. Unfortunately, very often in scholarly textbooks and research literature discussing papermaking, hydrogen bonding has been incorrectly used as a simple explanation for fiber–fiber interactions and paper strength. In a very recent review by Wohlert et al., an excellent overview was presented of the current knowledge of intermolecular interactions related to cellulose-based materials at different hierarchical scales, from oligomers to macroscopic fibers [44].

### 2.2. Revisiting Cellulose—The Lindman Hypothesis

#### 2.2.1. Considerations and Implications of Cellulose’s Dual Properties

The insolubility of cellulose in water is, in many publications, considered as a result of the complex hydrogen bonding network [45,46]. Since the general view of cellulose in the past suggested the dissolution of the polymer by breaking the cellulose–cellulose hydrogen bonds, it implied that the key to increasing cellulose’s solubility is to find a solvent that effectively disrupts the interchain hydrogen bonding in cellulose. Thus, the argued explanation for cellulose solubility in certain solvents, such as ILs, is that they “break” the hydrogen bonds. Moreover, in a somewhat imprecise way, some authors also refer to the crystallinity of cellulose as a contributing factor in its insolubility [47,48]. In general, polymer solubility is dependent on a balance between entropy, which drives solubility, and enthalpy, which opposes solubility. Entropy contributions are of different types, such as translational (determined by polymer molecular weight), configurational (determined by polymer flexibility) and counterion entropy (ionic polymers). Therefore, low-molecular-weight polymers are more soluble than high-molecular-weight ones, flexible ones are more soluble than stiff ones and ionic ones are more soluble than non-ionic ones.

Regarding the interactions determining the enthalpy, the strong hydrogen bonding between cellulose molecules has been emphasized in the literature. It is true that there are strong hydrogen bonds between cellulose molecules, but it has often been forgotten that, on dissolution in water, these are replaced by cellulose–water hydrogen bonds, which are equally as strong (ca. 5 kcal/mole) [49]. Hydrogen bonding can, therefore, not explain the low aqueous solubility. In fact, solute–solute hydrogen bonding in an aqueous solution is not expected to drive association or cause insolubility. Water is a highly hydrogen-bonded solvent. On the introduction of a solute, there is the formation of “cavities” in water. If the solute is nonpolar, there is a large opposing force for solubility, since cavity formation leads to a loss of water–water hydrogen bonding. There is partial compensation, especially at lower temperatures, because of the “structuring” of water around the co-solute; this leads to anomalously high solubility at low temperatures, as clearly manifested in the non-monotonic variation in many properties around room temperature [50]. On the association of two nonpolar molecules in water, “hydrophobic association”, there is a reversal of this process and a considerable gain in free energy due to the release of water molecules to the bulk (increasing the entropic contribution) and formation of water–water hydrogen bonds.

If cellulose is argued to be amphiphilic, its solubility and association behavior in water should be significantly affected by co-solutes known to eliminate/weaken hydrophobic interactions. In this respect, urea is well known to eliminate hydrophobic association in protein denaturation and surfactant demicellization, and surfactants have significant effects on cellulose solubility [46,51,52,53]. A general property of amphiphilic compounds is to migrate to interfaces, a tendency particularly observable in aqueous systems [54]. Further consequences are to self-assemble—for many polymers, this results in in gel formation—and to associate with other amphiphilic compounds, such as surfactants, polar lipids and block copolymers. 

Close examination of the crystal structure of cellulose reveals that there is clear spatial segregation of C-H and O-H bonds, thus leading to both nonpolar and polar regions and suggesting distinct amphiphilicity [55,56,57]. It can be noted that other polyglucoses, such as cyclodextrins and amylose, also display distinctly nonpolar domains and interact strongly with both polar and nonpolar molecules [58].

#### 2.2.2. Response from the Scientific Community on Lindman’s Dispute

The Lindman hypothesis [2] was debated by some of the most well-known cellulose scientists in a follow-up article [3]. The overall conclusion from his peers can be summarized by the following quote: “The general perspective of cellulose as a polymer in which intermolecular stress transfer involves more than hydrogen bonds. Hydrophobic and amphiphilic behaviors have been acknowledged for some time but may have been underestimated in conventional considerations of structure, solubility, etc. Ever since the discovery of hydrogen bonds, there has been a tendency to over-exaggerate their importance in determining the solid-state structure. The energy of a hydrogen bond is much more than the van der Waals energy of attraction between say C–H groups, but we must remember that there are a lot of C–H groups in cellulose.”

From the biosynthesis perspective, if it takes place in discrete steps in time and space, then these findings could reflect the structural inhomogeneities, which could lead us to better understand how to formulate more efficient cellulose solvents. Some other compounds, e.g., carboxymethylcellulose, have a very distinct and dramatic role, inhibiting higher-order aggregations in cellulose’s ribbon structure [59]. This would indicate that native agents co-secreted with cellulose could change and control cellulose’s crystallinity. Since living organisms create cellulose structures that are in fact different on some structural level, while being identical in chemical (molecular) structure, the properties and possible dissolution behavior would be different. The latter might be a consequence of the thermodynamically driven molecular aggregation process of cellulose chains, being influenced by the presence of heteropolysaccharides and proteins during the biosynthesis.

## 3. Recent Progress in the Scientific Understanding of Cellulose

**Figure 3:**
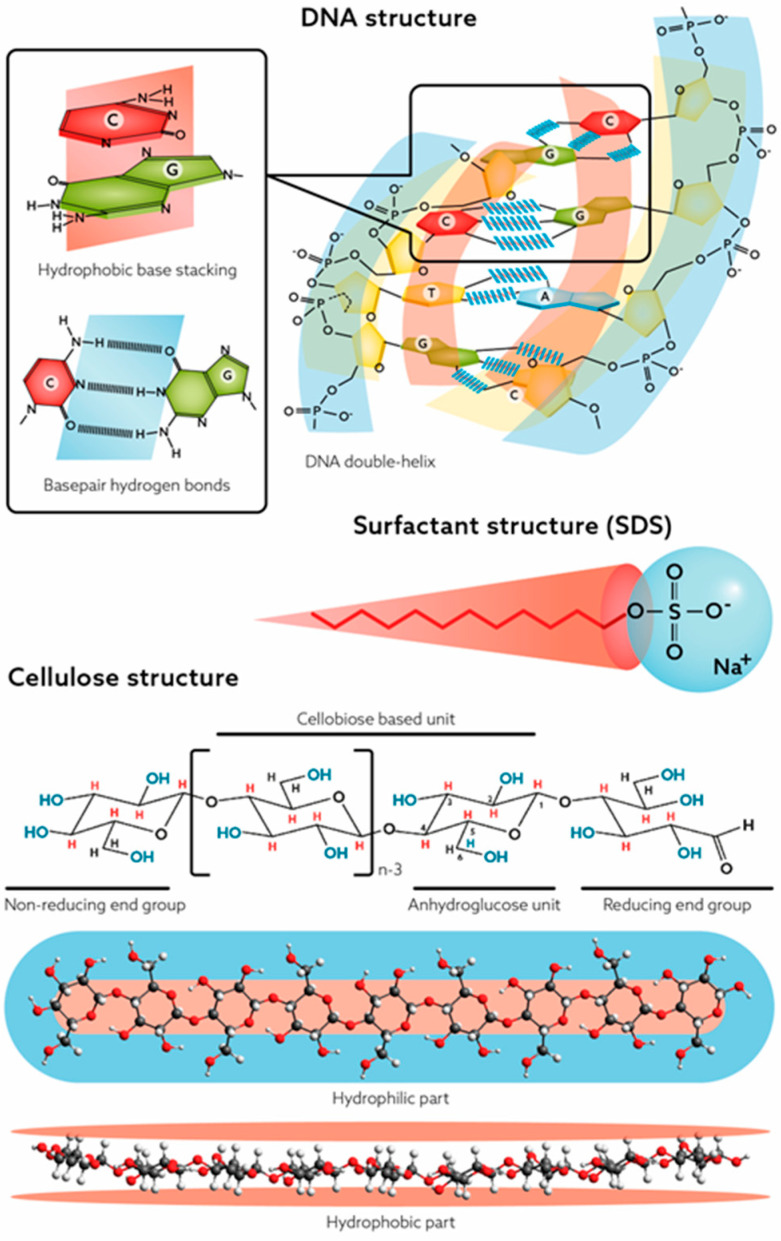
Amphiphilic nature of DNA, cellulose and surfactants. Adapted and reprinted from [61] with permission from Cambridge University Press, © 2023.

The previous lack of discussion and general acceptance of cellulose as being an amphiphilic molecule is understandable. The main explanation for this is that synthetic amphiphilic polymers, such as block and graft copolymers, have been more commonly produced in recent decades. Moreover, the perception of biomacromolecular amphiphilicity and the importance of hydrophobic interactions in biological macromolecular systems have been very limited and neglected. However, proteins and lipopolysaccharides are good and accepted examples of biological macromolecules possessing amphiphilic properties. The secondary structure in proteins is determined by a balance between hydrophilic and hydrophobic interactions. Recently, the importance of hydrophobic interactions has also received considerable attention in the field of biology regarding liquid–liquid phase separation [60]. Nevertheless, in DNA, where the double helix structure of DNA owes its stability to hydrophobic interactions, as well as in cellulose, the role of amphiphilicity has not been properly considered [61,62]. Figure 3 illustrates the physicochemical and structural characteristics behind the amphiphilic nature of the two biopolymers.

### 3.1. Theoretical Considerations of Molecular Cellulose

A large body of computer simulations has substantially contributed to the understanding of the origin of hydrophobic interactions, ranging from studies of the methane–water system [63] to micelle formation [64], peptide interactions [65] and DNA double-helix stability [66]. A particularly interesting study investigated the effect of salt in the methane–water system and concluded that “the number of broken H-bonds is significantly larger in the presence of salt and should contribute to an increase in the free energy of dissolution, and hence to a lowering of the solubility and an increase in the hydrophobic interaction” [67]. For cellulose explicitly, Miyamoto et al. investigated the structural reorganization of two different types of molecular sheets derived from the cellulose II crystal by using molecular dynamics (MD) simulations to distinguish the initial structure of the cellulose crystal during its regeneration from the solution [57]. The molecular sheet formed after a one-nanosecond simulation by van der Waals forces along the (1–10) crystal plane did not alter its structure in an aqueous environment, while the one formed by hydrogen bonds along the (110) crystal plane changed into a van der Waals-associated molecular sheet. The two structures showed substantial similarities, such as the high occupancy of intramolecular hydrogen bonds between O3^H^ and O5 of over 0.75, few intermolecular hydrogen bonds and the high occurrence of hydrogen bonding with water.

It is observed that the van der Waals-associated molecular sheet in the solution can be the initial structure of the cellulose crystal formed after the convergence of the two structures into one. The results imply that the van der Waals-associated molecular sheet becomes stable in a water environment with its hydrophobic interior and hydrophilic periphery. Interestingly, and consequently, in a benzene environment, a hydrogen-bonded molecular sheet is preferred, which is also expected to be the initial structure formed in benzene.

Bergenstråhle et al. used MD simulations to calculate the potentials of mean force for the separation of short cellooligomers in an aqueous solution as a means of estimating the contributions of hydrophobic stacking and hydrogen bonding to the insolubility of crystalline cellulose. The outcome of the study indicated that both hydrophobic association and hydrogen bonding favor the cellulose crystal, which makes it less surprising that cellulose is so insoluble. However, the contribution of H-bonding to cellulose’s insolubility in an aqueous environment was an order of magnitude smaller than the hydrophobic solvation energies [68].

Nishiyama studied the molecular interactions in nanocellulose assembly. The contribution of hydrogen bonds and the London dispersion force in the cohesion of cellulose was evaluated and discussed considering features, such as the structure, spectroscopic data, empirical molecular modeling parameters and the thermodynamic data of analogue molecules. The London dispersion interaction was estimated from empirical attraction terms in molecular modeling by simple integration over all components. The short-range interaction was found to be dominant for the cohesion of cellulose and equivalent to compression of 3 GPa. Because of the reduced number of hydroxyl groups in cellulose compared to simple alcohols, the London dispersion force provides the main contribution to intermolecular cohesion in nanocelluloses [69].

### 3.2. Dissolution of Cellulose

Since cellulose does not melt, dissolution is a required step for its feasible processing into different shapes and forms, such as fibers, films, particles, hydrogels and emulsions, among many others. The dissolution of cellulose is certainly not trivial, as the most efficient solvents are remarkably different in nature and thus the understanding of the delicate balance between the different interactions involved becomes difficult to rationalize, but it is very important for further solvent development and improvement. Nowadays, it is reasonable to state that, since the work of Prof. Lindman, the cellulose community is more aware of the decisive role of hydrophobic interactions in cellulose dissolution and regeneration in aqueous media. The mainstream view that H-bonds are the sole argument to explain cellulose behavior is now considered clearly overstated and the recent literature clearly supports this [70].

The amphiphilic nature of cellulose, the hydrophobic interactions and H-bonding’s role in dissolution and regeneration, the polyelectrolyte behavior at an extreme pH and the role of crystallinity were some of the critical aspects discussed by Prof. Lindman. All these aspects are quite relevant in polymer solubility (in general) and in cellulose (in particular) and have led to important discussions and eventually contributed to the development of new solvents and the improvement of some of the existing ones [14,71]. Many of these studies were carried out at Coimbra University (Portugal) and at different research centers in Sweden, with the main goal of developing new aqueous-based solvent systems for cellulose dissolution and shaping into novel fibers for textile applications [51,72].

**Figure 4:**
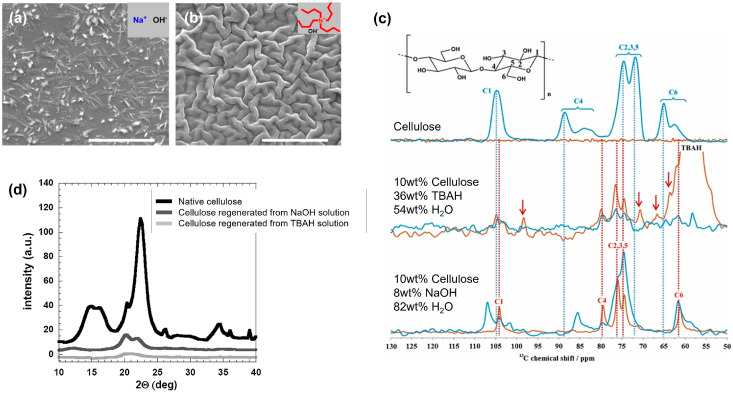
Scanning electron microscopy images of microcrystalline cellulose (MCC) dissolved in aqueous 8 wt% (2 M) NaOH (**a**) and in aqueous 40 wt% (1.54 M) tetrabutylammonium hydroxide (**b**). The scale bar represents 5 μm. PTssNMR of 10 wt% MCC in aqueous NaOH (**c**, bottom), 10 wt% MCC in aqueous TBAH (**c**, middle), MCC starting material (**c**, top) and X-ray diffraction patterns of regenerated cellulose from MCC dissolved in different solvents (**d**). (**a**–**d**) were adapted from [74], while (**c**) was adapted from [86] with permission from Elsevier, © 2023, and Royal Society of Chemistry, respectively, © 2023.

It is well known that cellulose can be dissolved in aqueous alkali at low temperatures [16], but the performance of the solvent regarding solubility, stability and rheological properties can be boosted by the addition of different additives (e.g., urea, thiourea, ZnO and amphiphilic molecules) [51]. Even lignin has been recently observed to enhance cellulose’s dissolution and stability [73]. Superior dissolution performance is also observed when small inorganic cations, such as sodium or lithium, are replaced by amphiphilic organic cations, such as tetrabutylammonium (TBA^+^) or tetrabutylphosphonium (TBP^+^) (Figure 4) [74]. These systems belong to the so-called family of “onium hydroxides”, which have shown a notable capacity to solubilize high concentrations of cellulose in relatively mild conditions [75,76,77].

The use of amphiphilic cations or species of intermediate polarity, such as urea or thiourea, typically enhances the stability of the cellulose dopes (delay of gelation), which is very important for many applications, such as fiber spinning [78,79]. This improvement in stability is mainly attributed to two factors: first, the amphiphilic-like or intermediate polarity species can avoid the re-aggregation of dissolved cellulose chains, driven by hydrophobic interactions [80,81,82,83,84], and secondly, a higher dissolution degree represents a lower fraction of undissolved crystallites that could potentially act as crosslinking points, as suggested by the work of Pereira et al. [79]. In this work, it was found that the formation of crystalline domains is due to the crystallization and precipitation of cellulose in aqueous NaOH, which eventually drives sample gelation.

Apart from the enhanced stability of cellulose solutions, the use of amphiphilic-like cations has been observed to improve dissolution and allows reaching a molecularly dissolved state of cellulose (Figure 4b). On the other hand, the use of inorganic counterparts, such as in NaOH-based systems (Figure 4a), often leads to incomplete dissolution. The understanding of the state of dissolution of cellulose in a certain solvent is not trivial and state-of-the-art techniques are of the utmost importance. In this regard, polarization transfer solid-state NMR (PTssNMR) has emerged as a very promising technique for the reliable and robust characterization of the solution state of cellulose [85] in different solvent systems, being capable to distinguish between dissolved and undissolved fractions and to characterize the cellulose polymorphs [86,87,88].

Returning to the alkaline-based systems containing inorganic or amphiphilic-like cations, PTssNMR confirmed the higher degree of dissolution in the latter case; an intense “insensitive nuclei enhancement by polarization transfer” (INEPT) signal coming from dissolved cellulose (red line in Figure 4c) is observed, while the “cross-polarization” (CP) signal, arising from the solid/undissolved cellulose fraction, is absent (blue line in Figure 4c). Due to the amphiphilic nature of organic cations, such as TBA^+^, it was anticipated that if their beneficial effect in cellulose dissolution is related to weakening hydrophobic interactions, then once the cations are hindered or removed from the solution, cellulose’s solubility should be compromised. This hypothesis was successfully tested using different cyclodextrins (CDs) as host agents to form complexes with TBA^+^ cations [89]. The formation of CD:TBA^+^ complexes was proven by NMR and the worsening of the solvent quality with CD addition was clearly observed from the polarized light microscopy and turbidimetry measurements. Overall, data support the hypothesis that the amphiphilic properties of TBA^+^ are determinant for the efficient dissolution of cellulose.

It is reasonable to assume that the dissolution state of a polymer such as cellulose may affect the way in which it arranges in a solution and how it organizes upon regeneration. Thus, the properties of the regenerated cellulose-based materials are expected to be deeply impacted by the dissolution level (i.e., molecular dissolution, colloidal aggregates). This is clearly seen in Figure 4d, where the regenerated cellulose is observed to be amorphous, in the case of previous dissolution with TBAH (aq), and crystalline (cellulose type II) for the materials regenerated from NaOH (aq). Results such as the ones previously discussed have inspired many scientists worldwide and contributed to the reevaluation of the role of the different interactions in cellulose’s behavior, even in nanocrystals [90,91].

The driving forces responsible for cellulose regeneration and the formation of crystalline domains have been evaluated by different authors [15]; for instance, Isobe et al. have demonstrated that the crystalline arrangement of cellulose is driven by hydrophobic interactions [92].

**Figure 5:**
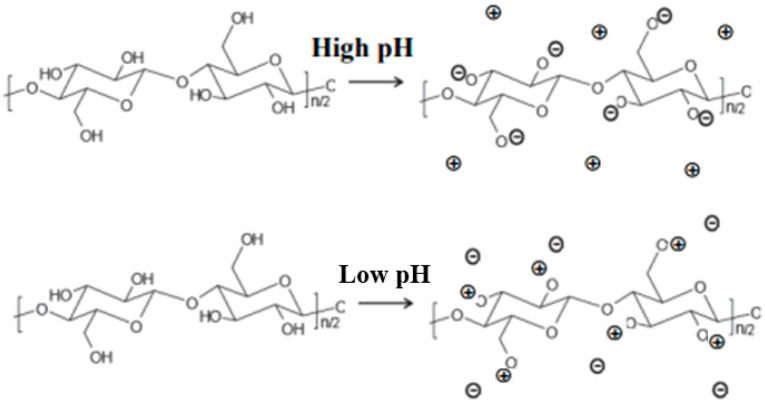
Conversion of neutral cellulose into a polyelectrolyte by pH change: schematic representation of the ionization of the hydroxyls of cellulose in extreme pH conditions, strongly alkali medium (high pH) and strongly acidic medium (low pH). Adapted from [71] with permission of DeGruyter, © 2023.

At this stage, it is clear that in order to develop novel solvents for cellulose or improve existing ones, different aspects need to be considered. One of these important, but often neglected, effects that has been already mentioned is related to the ionization of cellulose. Note that, since hydroxyls were thought to be solely engaged in the H-bonds, it was disregarded that they could also be involved in other phenomena, such as ionization. The role of the cellulose charge and the concomitant ion entropy effects should not be underestimated [14]. Contrary to the previously widely disseminated and accepted view focusing solely on the need to disrupt the H-bonding network in concentrated alkali/acids to promote cellulose dissolution, Prof. Lindman highlighted that the protonation/deprotonation of cellulose’s hydroxyl groups drives a nonionic polymer into a polyelectrolyte with enhanced solubility due to the counterion entropy [1] (Figure 5). This ionization effect was probed by Bialik et al., where electrophoretic NMR assays demonstrated that cellobiose may act as an acid with two dissociation steps, while MD simulations demonstrated that such charging of the cellulose chains in a solution prevented its aggregation (electrostatic repulsion among chains) and thus enhanced its solution stability [93].

Further NMR studies have shown that TBA^+^ cations bind to cellulose with ca. 1.2 TBA^+^ ions/AGU [94]. This was later supported by comprehensive scattering assays, where SAXS data were consistent with the formation of a sheath of bulky TBA^+^ ions solvating the cellulose molecules [95,96]. From a mechanistic point of view, the electrostatic interactions between the ionized cellulose molecules and the TBA^+^ cations are suggested to be the main driving forces [94]. Nevertheless, due to the above-mentioned amphiphilic properties of TBA^+^, it is reasonable to also anticipate a significant contribution from the hydrophobic effect regarding the favorable TBA^+^–cellulose interactions. These works strongly suggest that cellulose is, to a large extent, charged in a concentrated alkaline medium, an overlooked but relevant factor to rationalize its solubilization behavior.

Organic co-solvents, such as dimethyl sulfoxide (DMSO), have been observed to enhance cellulose’s dissolution, allowing the use of much less alkaline media [80,97,98,99]. Several solvent systems containing DMSO in their formulations have been successfully reported in the last decade [97,100,101,102,103,104,105,106,107,108]. DMSO, as a polar and aprotic co-solvent, displays major swelling properties for cellulose, thus facilitating the diffusion of solvent ions into the cores of cellulose fibrils [109]. DMSO is particularly efficient in decreasing the viscosity of different solvent systems, such as ILs, which also benefits the mass transport and dissolution efficiency [110,111]. Other authors also suggest that the addition of DMSO may enhance the solubility of cellulose in ionic liquid-based systems by weakening the electrostatic interactions among ions [112].

The work of Idström et al. is particularly interesting since cellulose–DMSO contacts were found to be three times longer than the DMSO–DMSO interactions [99]. Nevertheless, no clear role has been described for DMSO. Recently, Medronho et al. estimated that the fraction of “bound” molecules, Pb, of DMSO is ca. two times lower than the Pb of TBA^+^, which demonstrates the preferential interaction of TBA^+^ with cellulose [80]. The fact that the Pb values change less for DMSO than for TBA^+^ suggests the weaker adsorption of the former. As mentioned, DMSO facilitates cellulose dissolution, not only by tuning the solvent viscosity (enhancing mass transport) but also by solvating cellulose (here, the binding is not in the same sense as with the TBA^+^ ions), which facilitates further interaction between the TBA^+^ ions and cellulose. The highly polar character of the S-O bond in DMSO drives the overall negative charge density in the oxygen atom. On the other hand, the sulfur atom, despite displaying a positive charge density, carries a pair of non-bonding electrons [113]. Thus, both atoms are nucleophilic and not prone to interact with the negatively charged oxygen atoms of ionized cellulose. Furthermore, the hydrophobic features of the methyl groups in DMSO are expected to be less pronounced than those of the butyl groups in TBA^+^, which further substantiates the preferred interaction of cellulose for the latter.

As an emerging class of biomass solvents, deep eutectic solvents (DES) are interesting. Mixtures of natural bio-sourced cations and anions (natural DES or NADES), such as those obtained from natural organic acids, amino acids, non-nutritive sweeteners or natural compounds including choline or betaine, are particularly appealing due to their environmental benignity and relatively low cost when compared to, e.g., the synthetic ILs [114]. However, similarly to many ILs, the high viscosity of DES is a drawback for large-scale applications with cellulose. Moreover, the solubility of cellulose in DES is much lower than in most ILs [115,116].

### 3.3. Cellulose in Emulsions

**Figure 6:**
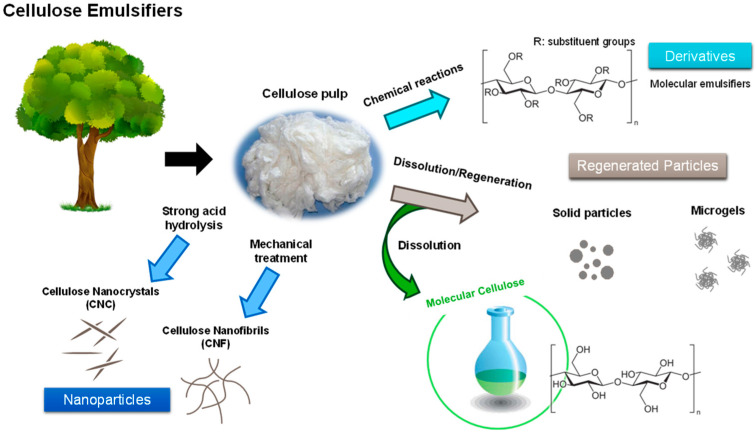
Cellulose emulsifiers and the different routes taken to achieve them.

Cellulose is a versatile source of natural emulsifiers and can be utilized as such over the whole hierarchical size range, from cellulose particles and gels to macromolecules; see Figure 6. The capability of all forms of cellulose to adsorb at oil–water interfaces and stabilize emulsions has been reported in the literature [117,118]. This phenomenon indirectly evidences the significance of the hydrophobic interactions between cellulose molecules and how they can affect the dissolution and regeneration of cellulose. Recently, it was observed that the stability of emulsions could be altered by tuning the solvent characteristics to achieve a more or less soluble cellulose in an aqueous environment. With a solvent such as TBAH (aq), an amphiphilic medium and good solvent for cellulose, it was not possible to form stable emulsions. On the other hand, with NaOH (aq), emulsions were formed and could be stabilized for a very long period, once cellulose was regenerated at the interface [73,119]. The same happens in the acidic solvent H_3_PO_4_ (aq) [120,121,122]. Another important observation was that molecular cellulose is indeed able to decrease the interfacial tension between an oil phase and the aqueous solvent phase, a property inherent to amphiphilic molecules, such as surfactants and polymeric surfactants [73,119,120,121,122,123,124,125]. This behavior is similar to what is observed for cellulose derivatives (e.g., MC and HPMC). Molecular dynamic simulations indicated that molecularly dispersed cellulose gradually assembles at the oil–water interface, eventually surrounding the oil droplet [123].

One means of producing emulsions using dissolved cellulose is by following a dissolution–emulsification “in situ” regeneration approach, where the oil is directly dispersed in the cellulose solution and regeneration takes place at the oil–water interface (in situ). A continuous-like coating is formed around the oil droplet, giving it a smooth appearance [118,121,124]. Another approach starting with a cellulose solution is by regenerating cellulose prior to emulsification, which can give Pickering emulsions of solid or soft cellulose particles (microgels), since the oil is either dispersed in a water suspension of cellulose particles or in a water suspension of cellulose microgels, respectively [117,118]. A fundamental difference between the two described approaches is related to the existence of dissolved cellulose during oil emulsification, which acts as a polymeric surfactant by decreasing the IFT and contributing to a reduction in the emulsion’s droplet size. Overall, the emulsions produced by both methods display very good stability against droplet coalescence, which can be attributed to the irreversible adsorption of cellulose onto the droplet surfaces [125].

Several researchers have also demonstrated the ability of cellulose particles to self-assemble at oil–water interfaces and to stabilize w/o emulsions without the aid of classical surfactants [126,127,128]. It is believed that the amphiphilic character of nanocellulose resides in its crystalline organization at the elementary “brick” level, and, thus, cellulose nanocrystals have both hydrophilic and hydrophobic edges that are preferentially wetted by water and oil phases, respectively. Cellulose’s stabilization of w/o emulsions is more challenging, but, a “hydrophobic” cellulose microgel has been recently developed, by regenerating cellulose in the presence of an oil [129]. This cellulose microgel is more easily dispersed in oil than water, and stable w/o emulsions can be formed.

### 3.4. Regeneration of Cellulose

Cellulose dissolved in a good solvent is solidified when its solvency is decreased by any means, and this can be attained in several ways. Most commonly, different non-solvents (antisolvents) are being used to obtain this efficiently. Regeneration can also be imposed by, e.g., the addition of different salts or a combination of an antisolvent and salt. Remarkably, regeneration can also be triggered by temperature changes, without the addition of any other substances. In an aqueous solvent system comprising alkali and urea, gelation occurs via a sol–gel transformation when increasing the temperature of the cellulose solution [78], similar to what is observed for nonionic polyoxyethylene systems. Depending on the nature of the solvent and the regeneration method, the solidified cellulose appears differently from the perspective of molecular ordering. There is no accepted evidence that native semi-crystalline cellulose that has undergone dissolution can be regenerated into its previous polymorph, cellulose I. Generally, cellulose II is formed, which is the most energetically favorable cellulose crystal structure. However, depending on the treatment, cellulose can also take the form of other polymorphs, e.g., cellulose III and cellulose IV [130].

**Figure 7:**
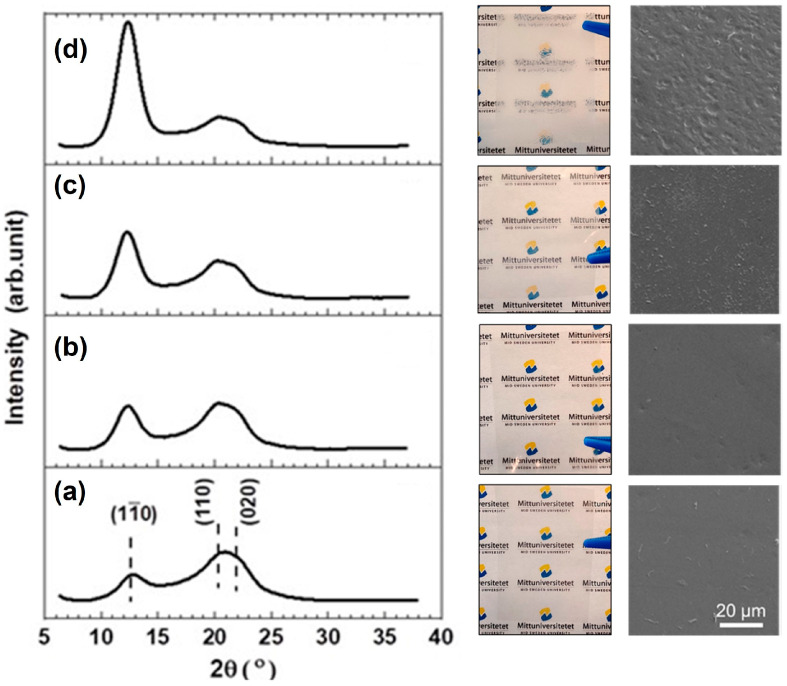
To the left, X-ray diffraction (XRD) patterns of the cellulose II films prepared with (**a**) methanol, (**b**) ethanol, (**c**) n-propanol and (**d**) n-pentanol. The intensity scales are identical. In the middle and to the right, photos and FE-SEM images of the corresponding films showing the differences in transparency and surface structure. Reprinted from ongoing work [133].

The above-mentioned methodologies can be utilized when dissolved cellulose undergoes regeneration into the solid state to control the degree of crystallinity. Since crystallinity affects many different material properties of the solidified cellulose, such as the surface morphology, transparency and haze, mechanical strength, density, water contact angle, moisture uptake and gas permeability [131,132], this is an interesting physical route to follow when tuning the cellulose II material. The opportunity behind this arises from a range of factors, where differences in cellulose–cellulose, cellulose–solvent and cellulose–antisolvent interactions exist and are important. Moreover, external force fields such as mass transport by flow and diffusion, which regulates the kinetics, and shear during solidification, which releases stress and imposes the relaxation of the polymer chains, strongly contribute to the final properties of the material. On a molecular level, cellulose’s amphiphilicity and the interplay between hydrophobic interactions and H-bonding regulates the finer details. The latter was observed in an XRD deconvolution analysis on solid films of cellulose II (Figure 7) obtained from alkali–urea-dissolved cellulose regenerated in alcohols of different hydrocarbon chain lengths [133]. In Figure 7, the expression of different crystallographic planes in cellulose II is observed as diffraction intensities for a certain reflection representing hydrophilic or hydrophobic unit cell planes [57,92,134].

## 4. Emerging Applications and Future Development

Global warming and the accumulation of microplastics in the oceans have driven research efforts toward developing bio-based and biodegradable materials to replace petroleum-based products. At the beginning of 2023, the European Polysaccharide Network Of Excellence (EPNOE) released the Research Roadmap 2040, a strategic document that summarizes the most relevant scientific questions to be answered for biomass and polysaccharides during the coming decades [135]. Cellulose, and particularly nanocellulose and regenerated cellulose, offers a sustainable alternative for the production of a variety of technical materials with a low carbon footprint, such as films for food packaging, membranes for filters, filaments for textiles and aerogels for insulation [136,137,138]. Yang et al. developed transparent and bendable cellulose films from alkali–urea systems, with high gas barrier properties [132] and high water repellency [139], suitable for food packaging. Composite films developed by Wu et al., based on cellulose, starch and lignin and dissolved in IL, showed good thermal stability, strong mechanical properties and low gas permeability [140]. By blending cellulose with chitosan and dissolving it in NMMO, high-strength films with natural antibacterial properties were produced [141]. Cellulose-based films with such properties could be beneficial for, e.g., food packaging and wound dressing. Similarly, in textile production, regenerated cellulose–chitosan composite fibers would be expected to give textile materials with antibacterial properties.

Recently, a promising breakthrough in IL development for the dissolution of cellulose was achieved by research groups in Finland, led by Profs. Herbert Sixta and Ilkka Kilpeläinen [142]. The methodology was developed into a production process and is currently being commercialized. In 2022, Prof. Sixta and Prof. Kilpeläinen were awarded the Marcus Wallenberg Prize for the development of high-performance textile fibers based on different qualities of wood pulp. This innovation builds on the design and synthesis of novel superbase ILs (1,5-diaza-bicyclo[4.3.0]non-5-enium acetate, [DBNH][OAc]) that enable the efficient dissolution of wood pulp in high concentrations and at a viscosity level suitable for a spinning dope. This spinning dope can then be spun into high-quality regenerated cellulose fibers for the textile industry [143].

### 4.1. Nanocellulose in Material Design

**Figure 8:**
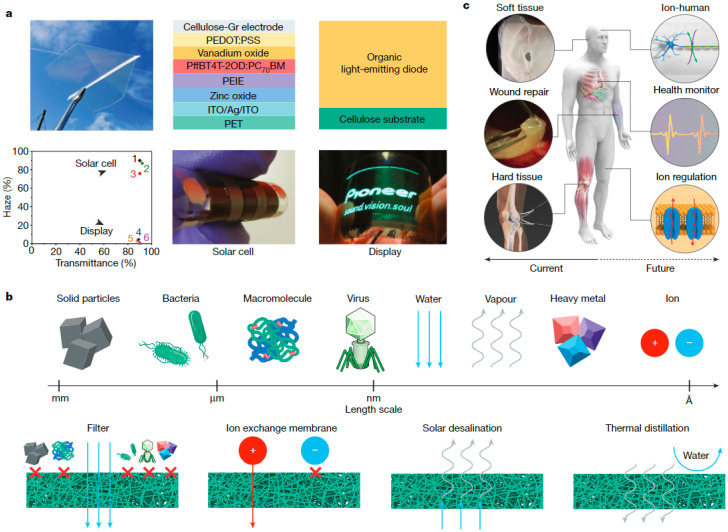
Fibrillated cellulose for far-term technologies. (**a**) Nanopaper for optoelectronics. Left, a photograph of transparent nanopaper (**top**) and the optical properties (**bottom**) of several selective nanopapers showing high optical transmittance and tunable transmittance haze. Middle, a layer diagram (**top**) and photograph (**bottom**) of a nanopaper-based solar cell. Right, a layer diagram (**top**) and photograph (**bottom**) of an organic light-emitting diode display. Gr, graphene; PEDOT:PSS, poly(3,4-ethylenedioxythiophene) polystyrene sulfonate; PEIE, polyethylenimine ethoxylated; PET, polyethylene terephthalate. (**b**) Selective transport of multiscale mass (from solids to ions) across different length scales in various fibrillated cellulose membranes for filtration, ion selectivity, solar desalination and thermally efficient distillation. (**c**) Fibrillated cellulose soft gels for bio-applications, including wound repair, soft and hard tissue engineering, ion regulation, the human (ion)–machine (electron) interface and health monitoring. Adapted from [151] with permission from Springer, © 2023.

The first isolation of nanocellulose occurred in the mid-20th century [144], while the advances in utilizing nanocellulose as a building block in developing new functional materials were only recognized at the beginning of the 21st century [145], when the more effective approaches for the isolation of nanocellulose with enhanced colloidal properties (charge-driven) were introduced [146]. In general, the term nanocellulose is considered for cellulosic particles with varying aspect ratios and at least one dimension in the nanoscale, namely cellulose nanofibrils (CNFs), cellulose nanocrystals (CNCs) and bacterial cellulose (BC) [147]. CNFs and CNCs are isolated from fibril aggregates by chemical and/or (sono)mechanical defibrillation (top-down approach), while BC can be obtained by bacterial oxidative fermentation (bottom-up approach) [147,148]. Although all types of nanocellulose have similar chemical compositions and structures (cellulose I), depending on the preparation protocol and origin, variations in properties such as the morphology, particle size and crystallinity will influence the overall properties and targeted applications [147,148,149]. As summarized in several reviews [146,147,148,149,150], nanocellulose’s properties, such as the high specific area and aspect ratio, high strength and high content of surface hydroxyl groups, make it a candidate for many potential applications, e.g., in energy, electronics, sensing, biomedical devices, food, packaging, optical devices, textiles, catalysts, insulation, decontamination and filtration applications, as exemplified in Figure 8 [151]. In addition, nanocellulose-based hydrogels have been shown to be very suitable for, e.g., electronic skin (e-skin) flexible electronics [152,153,154,155].

Considering the advances over the past twenty years, several challenges have been identified that hinder the transition of nanocellulose production and utilization to a large industrial scale. To overcome the difficulties, improvements in the production efficiency with environmentally friendly approaches and less energy-consuming processes to lower the cost are strongly recommended [146,147,148,149,150,156,157]. In this regard, quite recently, some researchers in the field have highlighted the importance of revisiting the fundamental understanding of nanocellulose–water interactions and the colloidal behavior of nanocelluloses in solving some of the challenges encountered [146,147]. A conclusion drawn from this was that nanocellulose has amphiphilic characteristics considering the properties and dynamics of a nanocellulose–water system, as concluded from the reported computational and experimental results on the distinction of hydrophilic (110) and hydrophobic (100) faces in cellulose I (hence nanocellulose) [147]. Regarding the nanocellulose dispersions, the intermolecular hydrogen bonding between the surface hydroxyl groups and van der Waals interactions between the hydrophobic sites in nanocellulose play different roles in the aggregation in water, organic solvents and polymer matrices.

### 4.2. Regenerated Cellulose with Added Functionalities

The term all-cellulose composite was first introduced by Nishino et al. [158]. By distinguishing the solubility of the matrix cellulose into the solvent from that of the cellulose fibers through pretreatment, all-cellulose composites were prepared. Shortly after this, Gindl and Keckes presented a study on nanocomposite films with different ratios of cellulose I and II, produced from partially dissolved microcrystalline cellulose in lithium chloride–*N*,*N*-dimethylacetamide [159]. Via the dissolution of cellulose in water-based solvents, such as the already described alkali–urea system, water-dispersible polymers and particles can be mixed with cellulose and co-regenerated into solid composite materials. Many possibilities to design novel materials that benefit from the inherent strength properties of cellulose, combined with the special features and functionalities of the additives, can be foreseen. The methodology has been utilized in several studies by Lina Zhang’s group in Wuhan [138]. Regenerated cellulose composite materials can be constructed from cellulose solutions in the presence of various organic and inorganic particles or polymers via blending with subsequent modification, often without any chemical reactions involved [160].

**Figure 9:**
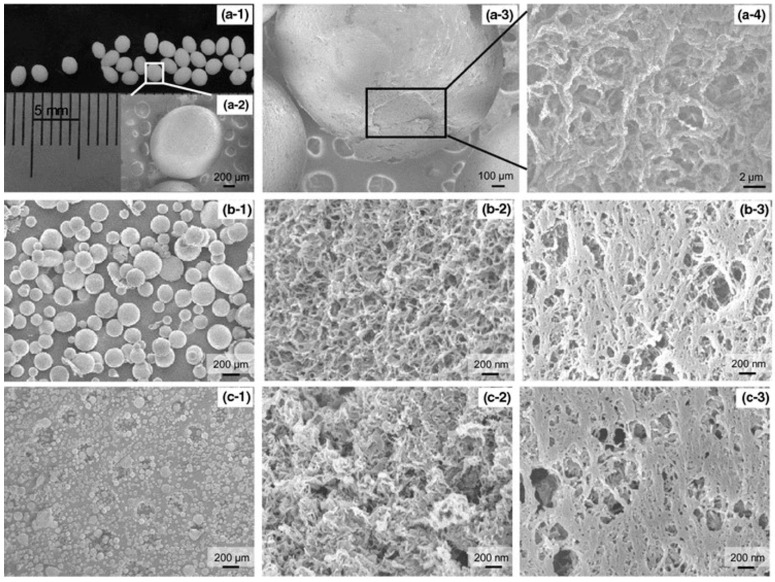
FE-SEM images at different magnification of the biocomposite particles prepared via different routes: (**a-1**–**a-4**) macroparticles, (**b-1**–**b-3**) chemically crosslinked microspheres, (**c-1**–**c-3**) non-crosslinked microspheres. Adapted from [166] with permission from Springer, © 2023.

Yang et al. prepared transparent and flexible cellulose–montmorillonite nanocomposite films by dissolving cellulose in a montmorillonite-dispersed LiOH–urea aqueous solution, followed by regeneration in acetone [161]. The high aspect ratio of the montmorillonite platelet and the formation of regular intercalated nanolayered structures in the cellulose matrix led to improvements in film properties. A cellulose–graphite oxide blended film was prepared by Han et al. by dissolving cellulose in a NaOH–urea–graphite oxide aqueous suspension, followed by coagulation in H_2_SO_4_ [162]. Composite films with 7.5 wt% graphite oxide immobilized in the cellulose matrix introduced a significant increase in the tensile strength and an improvement in the E-modulus. Morgado and Coma et al. and Almeida and Coma et al. studied bio-based films by dissolving and blending chitosan and cellulose in NaOH–thiourea, followed by film casting [163,164]. From their investigations, it was found that an increase in chitosan content led to higher tensile strength and strain before break. Similarly, Yang and Norgren et al. prepared cellulose–chitosan nanocomposite films via dissolution in alkali–urea and regeneration in ethanol/water [165]. The outcome of mechanical testing showed generally much better performance than in the previously mentioned studies, which could be due to the well-controlled drying conditions. However, the opposite trend in strength development was observed; upon increasing the chitosan content in the films, the tensile strength gradually decreased. In another study (Figure 9), cellulose–chitosan particles with different sizes and morphologies depending on the preparation technique were produced [166]. Particles in the millimeter scale, displaying core–shell structures, were prepared by dripping dissolved cellulose into an acetic solution of chitosan, resulting in instant co-regeneration. Two different approaches to obtain fully intermixed particles were applied by first mixing cellulose and chitosan solutions, with or without a crosslinking agent, followed by creating water-in-oil (w/o) emulsions in isooctane-Span^®^80. Thereafter, the sol–gel transition was triggered by increasing the temperature of the w/o emulsion and lowering the solubility of the biopolymer, to solidify the cellulose–chitosan droplets into micrometer-sized spherical nanocomposites. The two types of chemically or physically crosslinked microparticles obtained were thereafter washed, solvent-exchanged, freeze-dried from tert-butanol and thoroughly characterized.

Qi et al. reported on a sensor system prepared from cellulose dissolved in alkali–urea mixed with multi-walled carbon nanotubes (MWNT) and regenerated. MWNT–cellulose films displayed both flexible and conducting MWNT characteristics in an interval of 2–10 wt% MWNTs. Films with multifunctional sensing ability for the monitoring of stress–strain, temperature and humidity were obtained, with potential for electronic, magnetic, semiconducting and biotechnological applications and as water sensors [167,168].

Cellulose-based fibers from a combination of hydrophilic cellulose and hydrophobic polyaniline (PANI) were prepared to yield antistatic fibers [169,170]. PANI doped with acidic phosphate ester dissolved in cellulose–NaOH–urea at a low temperature gave a PANI–cellulose supra-molecular complex solution that was more stable than the cellulose solution itself, indicating good processability. Composite filament fibers were spun from the PANI–cellulose dope by wet spinning. No external doping was needed to achieve conductivity, presenting a viable method for antistatic fiber fabrication [171]. Fiber production via the electrospinning of single cellulose–NaOH–urea solutions has not been successful. However, by introducing high-molecular-weight polyethylene glycol (PEG), PEG–cellulose composite fibers, with a diameter of around 400 nm, could be electrospun [172].

Ye et al. recently presented a green route to the fabrication of strong and tough regenerated cellulose films [173]. The films comprised tightly stacked and long-range aligned cellulose nanofibers self-assembled from a cellulose solution in alkali–urea aqueous systems. Remarkable toughness of 41.1 MJ m^−3^ was reached in anisotropic nanofiber-structured cellulose films (ACFs). The well-aligned and densely packed cellulose nanofibers significantly decreased the interstitial spaces and minimized light scattering, giving ACFs with high optical clarity (91%), low haze (<3%) and birefringence behaviors. The simple methodology was argued to be scalable in fabricating high-strength, super tough and transparent cellulose films for emerging biodegradable next-generation packaging, flexible electronics and optoelectronic applications.

**Figure 10:**
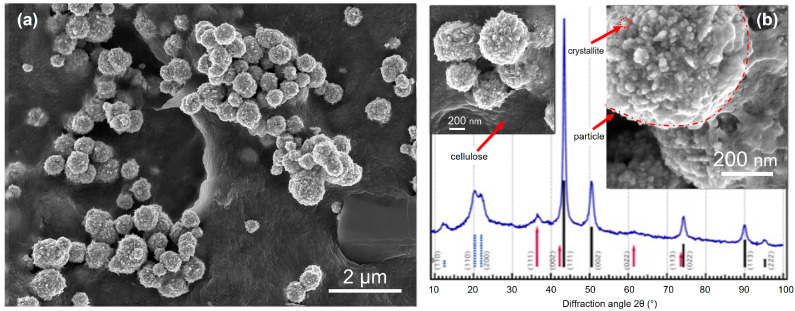
(**a**) FE-SEM images of synthesized hybrid material, Cu NPs in a regenerated cellulose network. (**b**) XRD pattern of the Cu NP–cellulose hybrid material. The positions of the expected Bragg peaks from copper (black lines), cellulose II (blue dotted lines) and cuprous oxide (red arrows) are marked and labeled with their respective Miller indices. The insets show FE-SEM images of the sample, highlighting the polycrystalline nature of the nanoparticles in the cellulose matrix. Adapted and reprinted from reference [174] with permission from Elsevier, © 2023.

An interesting approach to synthesize cellulose hybrid materials in situ has been described by Eivazihollagh et al. [174]. A facile method to procedure spherical copper nanoparticles (NPs), templated by a gelled cellulose II matrix under alkaline aqueous reaction conditions, was reported (Figure 10). The cellulose–copper hybrid material was prepared by the chemical reduction of chelated Cu^2+^ ions in a dispersion of alkali–urea-dissolved cellulose. The nucleation of the NPs was suggested to be initiated in the vicinity of the deprotonated hydroxyl groups in cellulose under highly alkaline conditions. Well-dispersed and spherical Cu/Cu_2_O NPs of a narrow size distribution decorating the cellulose were obtained. The hybrid material showed efficient antibacterial properties and its potential for catalytic and electronic applications was highlighted.

### 4.3. Cellulose in Electrical Applications

An electrical energy supply and utilization are essential for the functioning of society. In May 2021, the International Energy Agency (IEA) released its roadmap to global net-zero emissions, analyzing the implications of existing net zero pledges and showing a pathway to net zero emissions in electric energy production globally by 2050 [175]. In connection to this, a tremendous annual increase in off-grid electric energy storage and distribution is already appearing, and material solutions that can contribute to CO_2_ reduction, as with many other aspects of sustainability, are receiving increased attention scientifically and technically. Since the energy and climate crisis are two major challenges that we are facing, where the transition to green energy is urgently needed, this is an emerging field for cellulose-based materials. Cellulose can be utilized in many ways in energy storage and harvesting, and the whole value chain from the produced energy to the customer can thereby become more sustainable.

#### 4.3.1. Energy Storage—Supercapacitors and Batteries

Cellulose in different forms has been widely used as a separator, electrolyte, binder and dispersion agent in material for energy storage devices [176]. Nanocelluloses are most commonly used, especially CNFs and CNCs, due to their superior mechanical properties, flexibility, low cost, non-toxicity and appealing electrochemical properties.

**Figure 11:**
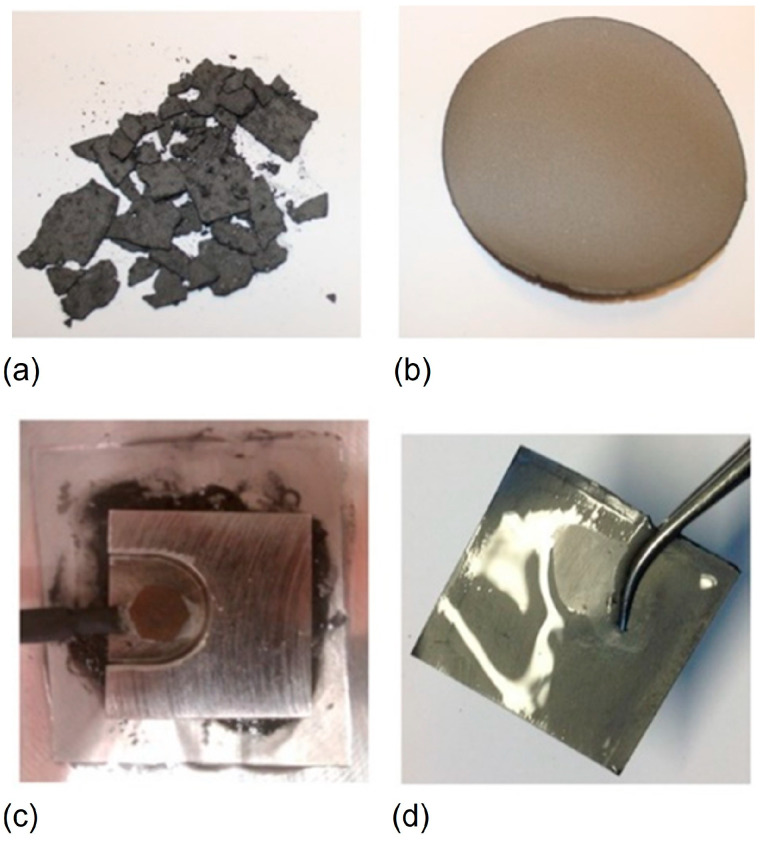
Mechanical stability and wet strength of battery–graphite films. (**a**) Battery–graphite film without NFC after light load. (**b**) Battery–graphite film with 10% NFC after light load. (**c**) Battery–graphite film without NFC. (**d**) Battery–graphite film with 10% NFC after operation in a SC. Reprinted from reference [181] with permission from Wiley-VCH Verlag GmbH & Co., © 2023.

Supercapacitors are considered one of the potential candidates in the domain of energy storage devices for future generations. There is a wide variety of applications, such as storing waste energy to power electric vehicles and other electronic systems and balancing the electric grid from fluctuations that arise from renewable energy sources such as solar and wind power. Supercapacitors have generated great interest due to their high power density, long cycle lifetime and fast charge–discharge [177,178]. A supercapacitor is usually constructed with two electrodes, with an ion-permeable separator between the electrodes, all soaked in an electrolyte. The electrodes often consist of carbon material, e.g., activated carbon [179], or graphene [180] as an active material and a binder that provides mechanical stability. CNF has been proven suitable as a binder without decreasing the electrical performance [181]. Figure 11 shows an example of how CNF enhances the mechanical stability of battery–graphite films.

**Figure 12:**
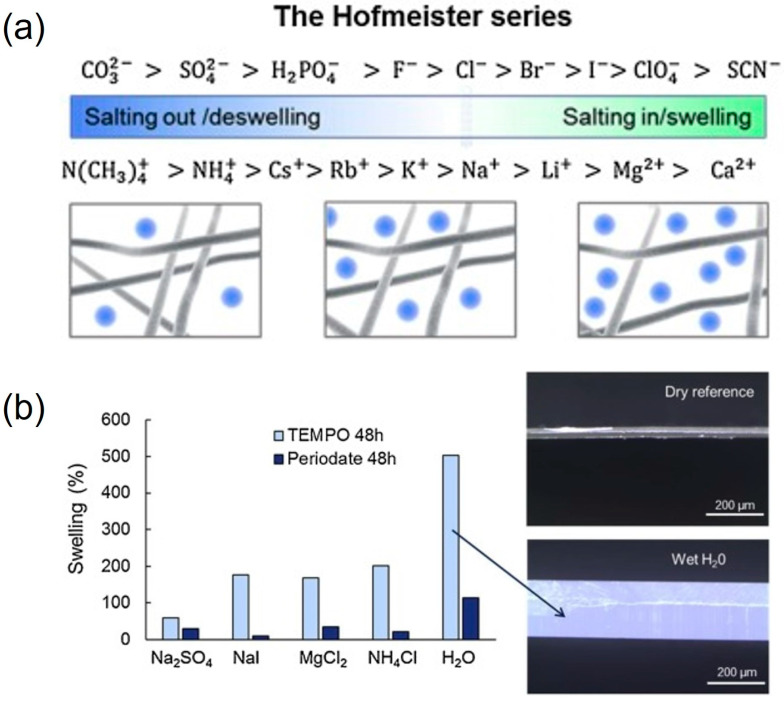
(**a**) Standard direct Hofmeister series for anions and cations based on precipitation studies of negatively charged proteins. To the right, ions have the highest stabilization power and increase swelling, whereas the left-hand side shows the opposite effect, for these specific systems. For the hydrophilic CNF system, the reversed series for cations is applicable. (**b**) Swelling behavior of TEMPO 48 h and periodate 48 h after soaking in different electrolytes/water for 20 min (**left**). All electrolytes are 1 M. Light microscopy cross-section images on TEMPO 48 h before and after soaking in water for 20 min (**right**). Adapted and reprinted from reference [188] with permission from Elsevier, © 2023.

Hajian et al. suggested that there is an association between nanocellulose and carbon nanomaterial, and the attraction increases with the surface charge of the cellulose [182]. CNC was also proven suitable to stabilize aqueous suspensions of carbon nanotubes [183]. The cellulose surface charges provide electrostatic stabilization of the cellulose–carbon complexes, preventing aggregation [184]. Between the electrodes, there is an ion-permeable separator, where cellulose-based materials have shown excellent properties, e.g., regenerated cellulose [185], paper [186], cellulose fibers [187] and nanocellulose [176]. The ion conductivity through CNF membranes was shown to depend on the choice of electrolyte, where swelling and deswelling of the network structure was observed based on the Hofmeister series [188] (Figure 12a). However, crosslinking via the periodate oxidation of cellulose inhibited the swelling (Figure 12b).

Batteries are power sources with high energy density, consisting of one or more electrochemical cells, with external connections that can be used to power electrical devices, such as electric cars and mobile phones. A battery consists of a negative electrode (anode), a positive electrode (cathode) and an electrolyte, and a separator is sometimes used to prevent short-circuits. In batteries, a binder is used to prevent electrode swelling and mechanical degradation and to protect the active material against the electrolyte, with maintained ion transport throughout the binder [189]. One of the challenges for high-energy-density devices, e.g., lithium-ion batteries, is that the active nanoparticles undergo a significant volume change during lithiation and delithiation. This results in mechanical stress on the binder surrounding the nanoparticles, which consequently leads to the degradation of the battery and decreased performance. Carboxymethyl cellulose (CMC) can be used as a binder, and it is capable of compensating for the volume change while maintaining stable performance [190]. Bridel et al. suggested that hydrogen bonding between the carboxyl group of CMC and the silica particles enabled self-healing during cycling [191]. CNC has been shown to be promising as a binder in Li-S batteries [192]. Separators are used as a physical barrier between the anode and cathode materials. It can also serve as an electrolyte reservoir for ion transport during the charging–discharging cycle. The separators are non-active electrochemical components; nevertheless, their features, such as their structural and physical properties, have a significant impact on battery performance [193]. Some key properties are related to the high ionic conductivity through the separator and low internal resistance. Different types of cellulose-based membranes have been used as separators, e.g., nanopapers made from CNF [194] and mesoporous membranes from CNC [195]. Polymer electrolytes, both gel and solid, are well-suited components in battery applications. Due to the local polymer chain relaxation, ion conductivity is enabled. Some examples of polysaccharides used are methyl cellulose [196] and ethyl cellulose [197]. Among supercapacitors, a hydrogel electrolyte produced from CNC, crosslinked with aluminum ions, exhibited superior ionic conductivity [198].

#### 4.3.2. Triboelectric Nanogenerators

Historically, due to its excellent dielectric properties, cellulose (paper) has been and still is widely used as an insulator material in high-power transformers [199,200]. Besides the traditional use of cellulose in the electric power industry, the need for small-scale distributed harvesting systems, especially suitable for sensor powering and wide-spread IoT solutions, is currently also growing. In this context, the triboelectric nanogenerator (TENG) is an interesting option with a work function that fits well in producing electricity from mechanical energy that is otherwise wasted [201]. The research on cellulose in the context of TENGs is, however, still in an early stage. TENGs comprise two separated dielectric surfaces electrostatically charged by frictional contacts between the surface layers. The transfer of electrons via an external circuit evens out the potential difference arising between the electrodes attached to the tribolayers [201,202]. TENGs can be designed and used as battery-less and self-sufficient power supplies for sensors and many other electronic devices needed for future sustainable solutions [203,204,205,206,207]. The vast majority of TENGs designed with organic tribolayers are based on synthetic polymers of questionable chemistry from a sustainable perspective. Owing to their great abundance, relative cheapness, renewability, non-toxicity and biodegradability as well as recyclability, the introduction of materials based on cellulose and several other biopolymers would be very well received [206].

**Figure 13:**
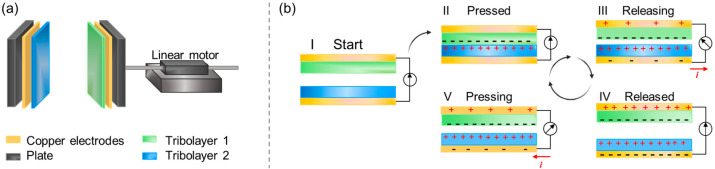
(**a**) Schematic illustration of the TENG setup and (**b**) the working mechanism following contact-separation mode. At (I) there are no charges on the tribolayer surfaces. Charge transfer occurs when the two tribolayers are pressed together, into physical contact (II). When the surfaces are separating (III), the static charges generate an electric field and induce a potential difference between the two electrodes, whereas electrons will flow in the external circuit from one electrode to the other in order to diminish the potential difference, creating a current, until fully released (IV). When the two layers are pressed into contact again (V), the charges are neutralized and the electrostatic field disappears, resulting in a current flowing in the opposite direction and producing an alternating current.

Cellulose was recently demonstrated as a very potent triboelectric material [208]. In a TENG comprising two slightly different cellulose II tribolayers operating in contact-separation mode, an output power density corresponding to 300 W m^−2^ could be reached. This is a striking result that shows that, once processed correctly, cellulose can fulfil the demands regarding the performance to replace fossil-based polymers in TENGs. Nevertheless, there is still a limited fundamental understanding of how the structural characteristics relate to the electric properties of cellulose and affect the output in triboelectric applications. However, in a newly published study, the dependence of cellulose’s physical structure on different length scales was elaborated more systematically with regard to the triboelectric performance [133]. Specifically, the effects of morphology and surface structural changes on the output power density were investigated. Native cellulose I was converted into cellulose II via dissolution in alkali–urea and regenerated in alcohols of varying aliphatic chain lengths. In Figure 13, an illustration of the TENG setup is shown and the working mechanism for the contact-separation mode is explained.

By utilizing triboelectric counter-layers with different mechanical softness, effects from both surface roughness and surface polarity could be followed in triboelectric power generation. The assigned peaks at 2θ 12.3°, 20.5° and 22° in the XRD diffractograms shown in Figure 7 correspond to the plane reflections (1–10), (110) and (020), respectively [209]. The intensity of the (1–10) diffraction peak increased with the increasing aliphatic chain length of the alcohols, and, correspondingly, the intensity of (110) and (020) was found to decrease. Isobe et al. reported similar findings regarding the surface and structural properties of cellulose hydrogels prepared from a LiOH–urea solvent with different alkyl alcohols as non-solvents [210]. Here, the hydrophilicity of the cellulose surface was assigned to the intensity of the (1–10) XRD reflection. This agrees very well with reported calculations of the surface energies of the different crystallographic planes, where (1–10) was shown to have the highest surface energy due to the frequency of exposed hydroxyl groups [56]. As previously discussed, the regeneration of cellulose from an aqueous alkali–urea system has been suggested to involve the hydrophobic stacking of glucopyranoside rings into monomolecular sheets, reflecting the (110) plane, followed by their mutual association by hydrogen bonding, reflecting the (1–10) and (020) planes, resulting in a hydrated form of the cellulose II polymorph [92,134]. MD simulations also show that the polarity of the coagulant controls the hydrophobic interactions between cellulose molecules during regeneration [57]. Moreover, this will induce the reorientation of the more hydrophilic parts of cellulose (hydroxyl groups) towards the film interface, which appears as an increase in the (1–10) reflection intensity, thereby producing more hydrophilic cellulose, which is further attributed to the low measured contact angles [57,92,134].

## 5. Concluding Remarks

Cellulose, growing on land and in the oceans, is the largest biomaterial resource on Earth and therefore an exceptionally important renewable raw material for humanity. With Prof. Lindman’s profound revisiting of the fundamentals of cellulose, scientific discussions of the interactions and mechanisms in cellulose systems have been facilitated. Cellulose’s amphiphilicity has been highlighted, and hydrogen bonding, which often has been used as the sole explanation for cellulose’s intramolecular interactions in aqueous systems, has been reconsidered theoretically as well as empirically in many papers. This has led to a deeper understanding of the hierarchical cellulose and its behavior, from molecules to macrofibers. This work paves the way to new thinking in all areas of cellulose technology, which is vital for the continuous and incremental optimization of different processes involving cellulose, as well as for the future development and design of novel cellulose materials to replace petroleum-based ones, with the aim of sustainable development.

## Data Availability

Not applicable.
